# Incidental Diagnosis of Uterine Perforation During Lower Segment Caesarean Section: A Rare Obstetric Complication

**DOI:** 10.7759/cureus.84223

**Published:** 2025-05-16

**Authors:** Pallavi Chauhan

**Affiliations:** 1 Obstetrics and Gynaecology, Balasaheb Thackrey Trauma Care Hospital, Mumbai, IND

**Keywords:** dilatation and curettage, missed abortion, perforation, polyglactin, premature rupture of membranes

## Abstract

This case report describes the incidental finding of uterine perforation during lower segment cesarean section (LSCS) in a 30-year-old woman, G2A1 with a history of a missed abortion. The antenatal period of the current pregnancy was uncomplicated. The patient presented at 39 weeks with complaints of leaking. A detailed examination revealed thick meconium, leading to the diagnosis of premature rupture of membranes. In response to the urgency posed by potential fetal distress, an emergency LSCS was performed. Post-delivery, an incidental intraoperative finding revealed a 2 cm circular uterine perforation at the fundus, actively bleeding. Swift surgical intervention utilizing absorbable suture polyglactin 2.0 achieved successful hemostasis, preventing potential severe complications such as postpartum endometritis and peritonitis. This case highlights the importance of vigilance and prompt intervention in managing unexpected obstetric complications during LSCS.

## Introduction

Uterine perforation, irrespective of the gravid status, is associated with significant morbidity and, in many cases, mortality as well [1]. It has been reported to occur at a very low incidence rate of approximately 0.8-6.4/1000 procedures [2]. This occurs as a result of rupture or breach in the uterine wall, which may result from trauma [3], surgical procedures such as cervical dilatation and curettage (D&C), cesarean section, or complications during labor [1,4]. The condition has been reported to result in significant complications such as injury to surrounding organs, hemorrhage, and infection [5]. Although a known risk factor, uterine perforation during lower segment cesarean section (LSCS) is rare and is often linked to previous uterine trauma or anatomical abnormalities. Timely recognition and surgical intervention in these cases are crucial in preventing severe outcomes and ensuring patient safety.

## Case presentation

A 30-year-old woman, G2A1L0 (second pregnancy with one previous abortion), registered at our hospital at 30.3 weeks of gestation. Her current pregnancy had been uneventful, with normal antenatal blood reports and scans. However, she had a history of a missed abortion at 7-8 weeks in March 2023, managed with D&C. The D&C was uneventful, and she conceived again shortly thereafter in April 2023. She recovered well post-procedure and continued with her pregnancy without complications.

At 39 weeks of gestation, the patient was admitted with complaints of leaking vaginally. Upon examination, the patient was vitally stable. On vaginal examination, thick meconium-stained amniotic fluid was identified with a fetal heart sound of 90-100 beats per minute, raising concerns of potential fetal distress. A diagnosis of G2A1 at 39 weeks of pregnancy complicated by premature rupture of membranes with meconium was made, necessitating an emergency LSCS.

**Figure 1:**
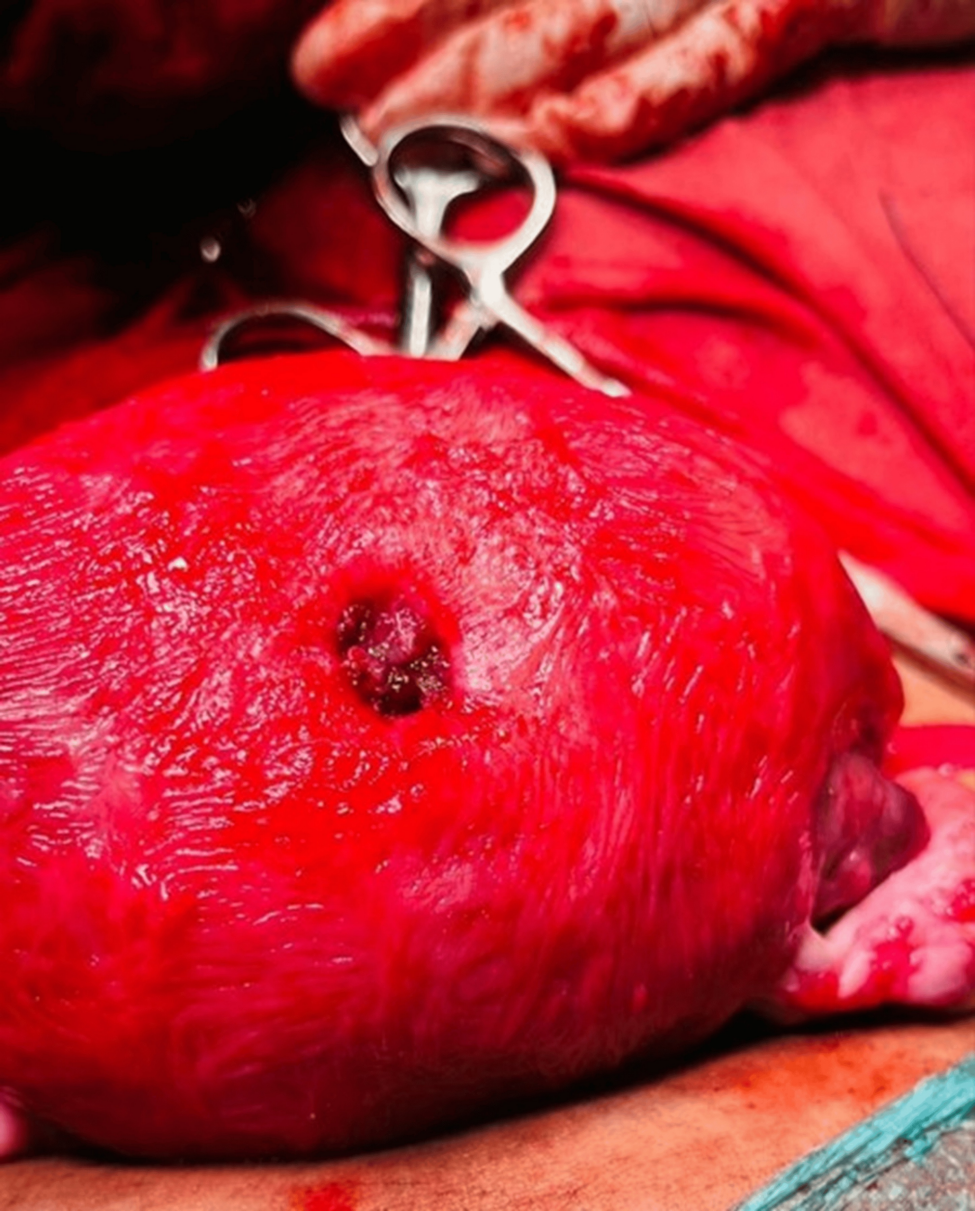
Circular uterine perforation

**Figure 2:**
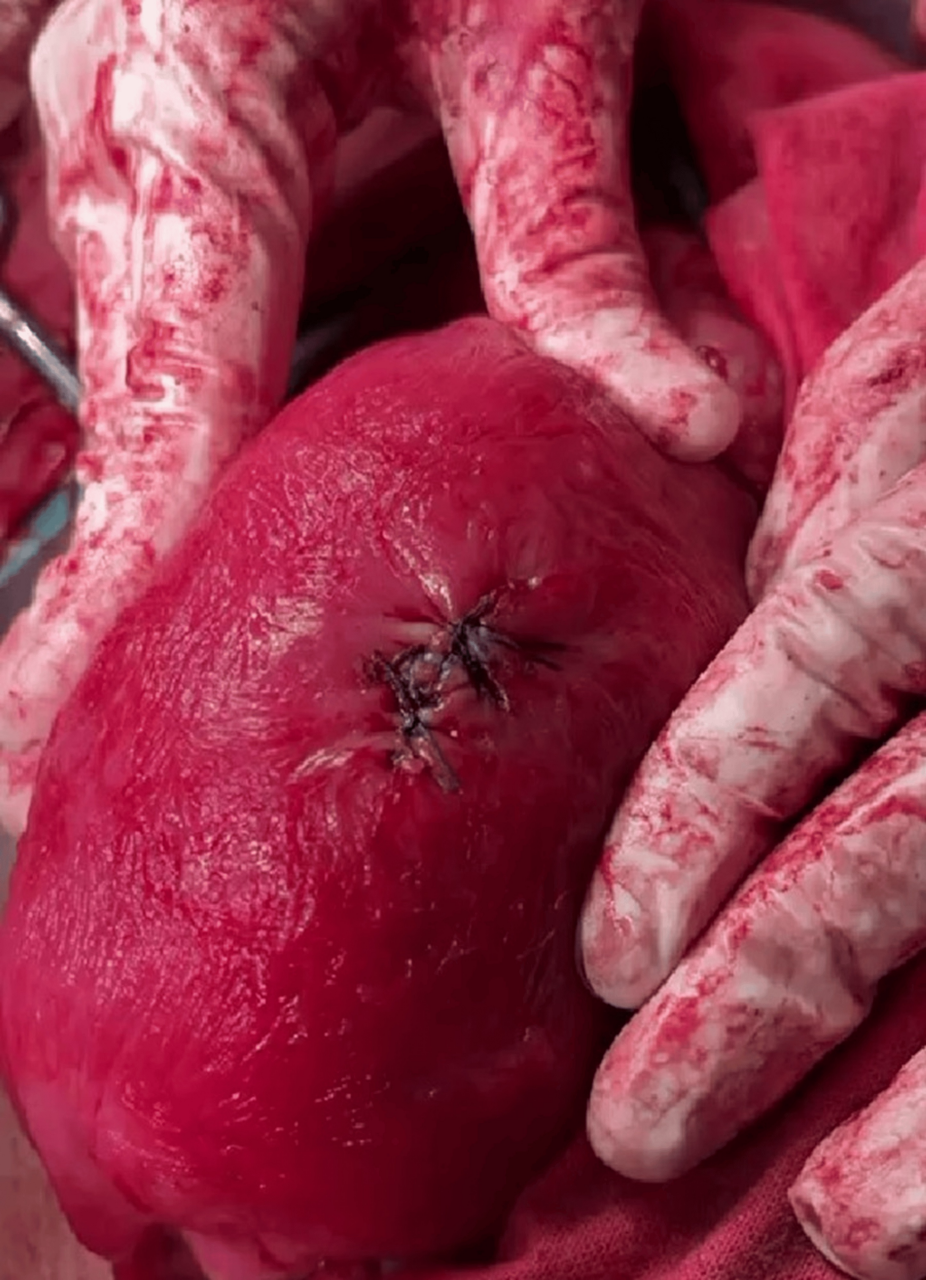
Perforation closed with absorbable suture polyglactin 2.0

During the LSCS, following the delivery of the baby and placenta, the uterus was exteriorized for examination. An incidental finding of a 2 cm circular perforation at the uterine fundus was noted, through which a finger could easily pass (Figure 1). The edges of the perforation were actively bleeding, requiring immediate surgical intervention. The perforation was closed using absorbable polyglactin 2.0 sutures in an interrupted manner, achieving successful hemostasis (Figure 2).

## Discussion

Uterine perforation is a potential complication of the intrauterine maneuvers used for evacuation or sampling of the endometrial cavity. Although rare, it is attributed to several maternal consequences [1]. Additionally, this iatrogenic condition, characterized by perforation and local destruction of the entire uterine wall, can significantly compromise future fertility [5,6]. However, uterine perforation following D&C can impact surrounding pelvic organs, potentially leading to their involvement or even causing them to be pulled or displaced into the uterine cavity [1]. The injury of the surrounding organs can sometimes lead to emergencies that require prompt medical intervention, potentially endangering the life of the patient [1,4]. The timely recognition and management of unexpected uterine perforation in our case were crucial to prevent serious complications such as uterine rupture, postpartum endometritis, and peritonitis.

A similar case was reported by Reed in 2003, highlighting a significant complication of uterine perforation, which can occur during D&C with long-term implications for subsequent pregnancies. They reported a fundal defect during LSCS, possibly caused by an unnoticed uterine perforation during a previous D&C procedure for a late first-trimester fetal death, underscoring the potential long-term complications of uterine injury [7].

Women with a history of uterine perforation should be carefully counselled about the potential for chronic defects. Such defects can affect uterine integrity and may lead to complications in subsequent pregnancies, including preterm rupture of membranes, abnormal labor, or increased risk of uterine rupture during delivery. Creinin and Chen have reported the potential long-term complications associated with fundal perforation following hysteroscopic resection of a uterine septum. They report a patient with term twin pregnancy and spontaneous rupture of membranes having a 7-cm defect in the uterine fundus during LSCS. The perforation was chronic in nature and was evident from the scarred edges and lack of bleeding [8].

A retrospective study by Shwarzman et al., examining the obstetric outcomes of 51 women diagnosed with uterine perforation, reported that most cases occurred during intra-uterine device insertion (76.5%), while a smaller proportion was associated with surgical procedures (23.5%) [9]. The study further mentions that adverse outcomes were relatively rare; however, one case of intrauterine fetal death due to fetal malformations and one instance of uterine rupture were reported, underscoring the potential risks associated with a history of uterine perforation.

The present case highlights the importance of thorough examination and vigilance during obstetric procedures, even in seemingly routine situations. The short time frame between miscarriage and subsequent pregnancy may not have given enough time to heal the uterine injury caused during D&C. Timely identification and management of such complications are essential for ensuring the well-being of both the mother and the newborn. Also, uterine perforation during LSCS necessitates prompt recognition and intervention to prevent severe complications. Factors contributing to perforation, such as previous uterine trauma, should be considered. Further research and analysis may be warranted to explore the potential risk factors associated with uterine perforation in subsequent pregnancies following a history of missed abortion and later conception. Additionally, this case underscores the significance of continuous monitoring and preparedness for unexpected complications in obstetric care.

## Conclusions

Examining the uterus after exteriorization is vital to identify abnormalities in shape, size, or defects such as old perforations. Patients with a history of D&C, difficult Hegar dilation, or operative hysteroscopy may have sustained known or undiagnosed uterine injuries, increasing their risk of rupture during pregnancy. This case underscores the importance of vigilance in obstetric procedures, particularly the unexpected occurrence of uterine perforation during LSCS, and the need for prompt surgical intervention. The probability of uterine rupture should be carefully considered when managing deliveries in patients with a history of uterine perforation. Patient counseling, awareness, and preparedness for such complications are essential to ensure optimal maternal and fetal outcomes.
